# Aspirin Use for the Primary Prevention of Myocardial Infarction Among Men in North Carolina, 2013

**DOI:** 10.5888/pcd12.150342

**Published:** 2015-11-19

**Authors:** Samuel Tchwenko, Eleanor Fleming, Geraldine S. Perry

**Affiliations:** Author Affiliations: Samuel Tchwenko, North Carolina Division of Public Health, Raleigh, North Carolina, and Gillings School of Global Public Health, University of North Carolina at Chapel Hill, Chapel Hill, North Carolina; Geraldine S. Perry, Epidemiology and Surveillance Branch, Division of Population Health, National Center for Chronic Disease Prevention and Health Promotion, Centers for Disease Control and Prevention, Atlanta, Georgia. Dr Fleming is also affiliated with the North Carolina Division of Public Health, Raleigh, North Carolina.

## Abstract

**Introduction:**

The US Preventive Services Task Force recommends aspirin use for men aged 45 to 79, when the potential benefit of preventing myocardial infarctions outweighs the potential harm of gastrointestinal hemorrhage. We determined prevalence and predictors of aspirin use for primary prevention of myocardial infarction vis-à-vis risk among men aged 45 to 79 in North Carolina.

**Methods:**

The study used data for men aged 45 to 79 without contraindications to aspirin use or a history of cardiovascular disease from the 2013 North Carolina Behavioral Risk Factor Surveillance System survey. Stratification by risk of myocardial infarction was based on history of diabetes, high cholesterol, high blood pressure, and smoking. Analyses were performed in Stata version 13.0 (StataCorp LP); survey commands were used to account for complex sampling design.

**Results:**

Most respondents, 74.2% (95% confidence interval [CI], 71.2%–77.0%), had at least one risk factor for myocardial infarction. Prevalence of aspirin use among respondents with risk factors was 44.8% (95% CI, 41.0–48.5) and was significantly higher than the prevalence among respondents without risk factors (prevalence ratio: 1.44 [95% CI, 1.17–1.78]). No significant linear dose (number of risk factors)–response (taking aspirin) relationship was found (*P *for trend = .25). Older age predicted (*P* = .03) aspirin use among respondents with at least one myocardial infarction risk factor.

**Conclusion:**

Most men aged 45 to 79 in North Carolina have at least one risk factor for myocardial infarction, but less than half use aspirin. Interventions aimed at boosting aspirin use are needed among at-risk men in North Carolina.

## Introduction

Myocardial infarction (MI) is responsible for 5.4% of all deaths among US men (1). In North Carolina, about 5.6% of men have had a heart attack and about 45 per 100,000 die from acute MI each year (2,3). Black non-Hispanic men in North Carolina are about 30% more likely to die and approximately 5% more likely to be hospitalized for MI than white non-Hispanic men.

Evidence supports aspirin use for primary prevention of cardiovascular disease (CVD) events, including MI (4,5). In 2009, the US Preventive Services Task Force (USPSTF) recommended aspirin for primary prevention of MI in men aged 45 to 79 years, when the potential benefit (reduction in MI) outweighs the potential harm of gastrointestinal hemorrhage (6). The US Department of Health and Human Services’ Million Hearts campaign promotes appropriate aspirin therapy for those who would benefit from its use as one component of the ABCS (aspirin use, blood pressure control, cholesterol management, and smoking cessation) of heart disease and stroke prevention (5).

Despite the USPSTF recommendation, physicians are underprescribing aspirin to patients (7), and high-risk patients are underusing aspirin for the primary prevention of MI (8–12). To our knowledge, no study has used national Behavioral Risk Factor Surveillance System (BRFSS) or North Carolina BRFSS data to assess aspirin use in the at-risk groups mentioned in the USPSTF recommendation. Our overall objective was to assess aspirin use among men in North Carolina for whom aspirin is potentially beneficial, according to the USPSTF recommendation, to 1) determine the prevalence of aspirin use among men aged 45 to 79 years; 2) assess aspirin use by number of risk factors for MI; and 3) identify predictors of aspirin use among men with risk factors for MI. This study updates the public health literature on aspirin use among subpopulations that may benefit from aspirin.

## Methods

The BRFSS is an annual standardized, state-based, random-digit–dial telephone survey of the noninstitutionalized US population and is described in detail elsewhere (13). To match the specifications of the 2009 USPSTF recommendation, we limited our study population to male respondents of the 2013 North Carolina BRFSS survey aged 45 to 79 years.

During the 2013 North Carolina BRFSS survey, some optional modules, including the cardiovascular health module, which asks about aspirin use, were administered to a random subsample (approximately half) of eligible respondents. The North Carolina State Center for Health Statistics uses this method (a split-sample questionnaire) to ensure data collection on multiple BRFSS optional modules and state-added questions of interest to public health programs while keeping interview length within acceptable limits (between 20 and 30 minutes) to minimize participant drop-off (unpublished data, NC State Center for Health Statistics, 2013). In 2013, the cardiovascular health module was administered to 931 of a possible 2,094 male BRFSS respondents aged 45 to 79. Given that 240 of the 931 eligible respondents would have been dropped from our study based on exclusion criteria (Appendix A) and the statistical limitations (ie, low precision of estimates, nonconvergence of models in regression analysis) of a small sample (691 respondents), we assessed the feasibility and validity of imputing response values for the cardiovascular health module for the 1,163 participants to whom the module was not administered. We verified that the distribution of all study covariates of interest did not differ significantly (within 5%) between respondents and nonrespondents of the cardiovascular health module (Appendix B) before proceeding with imputation. In other words, we tested and determined that the mechanism of missingness of our outcome was “missing at random” (14). This finding meant that we could either ignore the missing outcomes or impute the missing data and incur minimal bias. Covariates of interest were hypertension, diabetes, cholesterol, smoking, sex, age, race/ethnicity, education, health status, health insurance coverage, health access limitation due to cost, time since last medical checkup, and body mass index. Imputation allowed us to maintain an eligible study population of 2,094 respondents.

We excluded respondents with a self-reported contraindication to aspirin use or a history of any CVD to limit the scope of the study to aspirin use for primary prevention only. We considered participants who denied aspirin intake and responded either “yes, not stomach related” or “yes, stomach problems” to the cardiovascular health module question “Do you have a problem or health condition that makes taking aspirin unsafe for you?” to have a contraindication to aspirin use. We determined history of CVD by a yes response to at least one of the following items from the chronic health conditions section of the BRFSS core: 1) “Has a doctor or nurse, or other health professional ever told you that you had a heart attack also called a myocardial infarction?”; 2) “Has a doctor or nurse, or other health professional ever told you that you had angina or coronary heart disease?”; and 3) “Has a doctor or nurse, or other health professional ever told you that you had a stroke?”

**Figure 1:**
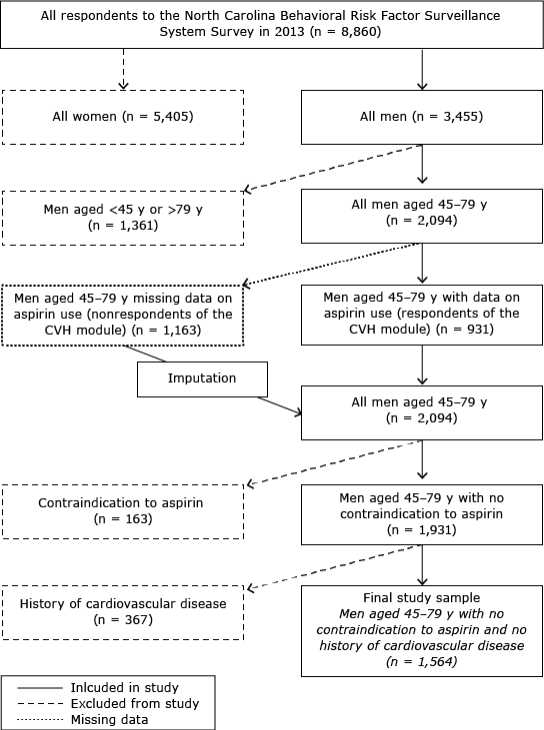
Selection of study participants, North Carolina, Behavioral Risk Factor Surveillance System, 2013. Abbreviation: CVH, cardiovascular health.

Therefore, of the initial 2,094 eligible participants, 163 were excluded because of a self-reported contraindication to aspirin use, and 367 participants were excluded because of a self-reported history of CVD, resulting in a final study sample of 1,564 respondents (Figure 1).

The primary outcome of interest, aspirin use, was ascertained by a yes response to the question “Do you take aspirin daily or every other day?” from the cardiovascular health module. The secondary outcome of interest, aspirin use for the prevention of MI, was ascertained by a yes response to the question “Do you take aspirin to reduce the chance of a heart attack?” from the cardiovascular health module.

Using responses to the BRFSS core, we stratified respondents’ potential risk of MI based on 4 modifiable risk factors: diabetes, high cholesterol, high blood pressure, and smoking. We selected these risk factors to match as much as possible the factors identified in the online Coronary Heart Disease (CHD) Risk Calculator from the Medical College of Wisconsin, which uses information from the Framingham Heart Study to determine 10-year risk of developing CHD (15,16). We performed hypothetical risk assessments for respondents of various ages in our sample using the CHD Risk Calculator and determined that the presence of any one of the 4 selected risk factors placed respondents in a risk stratum for which the benefits of aspirin may outweigh the risk of gastrointestinal bleeding, according to Figure 1 of the 2009 USPSTF recommendation statement (6). We also performed dose–response analysis and determined that prevalence of aspirin use did not vary with an increasing number of risk factors. Therefore, we dichotomized risk of MI and assumed that for participants who reported any one of the risk factors, the potential benefit of reducing MI risk outweighed the potential harm of gastrointestinal bleeding.

For univariate analysis, we used descriptive and summary statistics (means for continuous variables and proportions for categorical variables). For bivariate analysis, we used survey-adjusted *F* statistics as well as linear and log-binomial regression models to assess the relationship between aspirin use and risk factors and potential predictors. We considered as strong predictors all factors associated with aspirin use at a significance level of *P* < .05 during bivariate analyses and retained these factors for multivariate regression. Our analyses were performed using Stata version 13.0 (StataCorp LP) and survey (*svy*) commands to account for the complex sampling design of the BRFSS survey.

## Results

Most study participants (75.7%) were aged 45 to 64 years and either white non-Hispanic (72.7%) or black non-Hispanic (19.3%); 85% of respondents reported having health insurance coverage, and 74.5% reported having seen a health care provider for a routine medical checkup within the previous 12 months (Table 1).

**Table 1:** Aspirin Use for Prevention of Myocardial Infarction Among Men Aged 45 to 79: General Characteristics of Study Population (n = 1,564), Behavioral Risk Factor Surveillance System, North Carolina, 2013

Characteristic	n	Weighted % (95% CI)
**Age, y**		
45–54	483	42.4 (39.1–45.7)
55–64	555	33.3 (30.4–36.3)
65–74	408	19.2 (17.0–21.6)
75–79	118	5.1 (4.1–6.4)
**Race/ethnicity**		
White non-Hispanic	1,111	72.7 (69.7–75.6)
Black non-Hispanic	263	19.3 (16.7–22.2)
Hispanic	53	4.0 (2.9–5.3)
Other non-Hispanic	120	4.0 (3.0–5.3)
**Education**		
Less than high school	192	15.6 (13.1–18.4)
High school	452	29.4 (26.6–32.5)
More than high school	918	55.0 (51.7–58.2)
**General health status**		
Fair or poor	309	19.6 (17.2–22.3)
Good, very good or excellent	1,248	80.4 (77.7–82.8)
**Health insurance coverage**		
No coverage at all	193	15.0 (12.7–17.5)
Some kind of coverage	1,365	85.0 (82.4–87.3)
**Limited health care access due to cost**		
No	1,385	88.1 (85.8–90.0)
Yes	174	11.9 (10.0–14.2)
**Last routine medical checkup**		
A year or more ago	342	25.5 (22.6–28.5)
Within the last 12 months	1,206	74.5 (71.5–77.4)
**Body mass index (kg/m^2^)**		
Normal weight (18.0–24.9)	317	20.4 (17.9–23.2)
Overweight (25.0–29.9)	715	47.5 (44.2–50.8)
Obese (≥30.0)	486	32.1 (29.1–35.3)
**Number of myocardial infarction risk factors^a^**		
0	382	25.8 (23.0–28.8)
1	524	33.8 (30.8–36.9)
2	451	28.2 (25.4–31.2)
3 or 4	207	12.2 (10.2–14.5)

Abbreviation: CI, confidence interval.

a Risk factors were hypertension, diabetes, smoking, and high cholesterol.

Almost 75% of respondents had one or more risk factors for MI: 33.8% had one risk factor, 28.2% had 2 risk factors, and 12.2% had 3 or 4 risk factors (Table 1). Forty-eight percent of respondents had hypertension, 48.3% had high cholesterol, 21.1% were current smokers, and 15.5% had diabetes. The prevalence of aspirin use in the overall study sample was 41.2% (95% CI, 38.1%–44.4%) (Table 2). Of those who were taking aspirin, 87.8% (95% CI, 83.6%–91.0%) said they were taking aspirin to prevent heart attacks or MI. Except for current smoking, risk factors for MI were independently associated with higher prevalence of aspirin use (Table 2).

**Table 2:** Prevalence of Aspirin Use and Association Between Aspirin Use and Myocardial Infarction Risk Factors Among Men Aged 45 to 79 Years in North Carolina (n = 1,564), BRFSS, 2013

Subgroup	n^a^	Weighted^b^ Prevalence (%) of Aspirin Use (95% CI)	Prevalence Ratio (95% CI)
**Overall sample**	1,564	41.2 (38.1–44.4)	—
**Risk of myocardial infarction^c^**			
Low risk	382	31.0 (25.0–37.0)	1.0 [Reference]
High risk	1,182	44.8 (41.0–48.5)	1.44 (1.17–1.78)
**History of hypertension**			
No	766	36.2 (31.8–40.6)	1.0 [Reference]
Yes	798	46.6 (42.0–51.2)	1.29 (1.10–1.50)
**History of diabetes**			
No	1,295	39.1 (35.7–42.6)	1.0 [Reference]
Yes	267	52.2 (44.1–60.4)	1.33 (1.12–1.59)
**Current smoking**			
No	1,246	43.0 (39.4–46.6)	1.0 [Reference]
Yes	296	36.0 (28.9–43.1)	0.84 (0.68–1.04)
**History of high cholesterol**			
No	711	37.2 (32.6–41.8)	1.0 [Reference]
Yes	712	46.7 (41.9–51.6)	1.26 (1.07–1.48)

Abbreviations: BRFSS, Behavioral Risk Factor Surveillance System; CI, confidence interval.

a Totals for some subcategories do not add to overall sample size (1,564) because of missing data.

b Prevalence estimates are weighted to the overall population of men aged 45 to 79 in North Carolina using precalculated complex weight variables that are included in the BRFSS dataset.

c Level of risk was dichotomized as high (≥1 risk factor) or low (no risk factors). Risk factors were hypertension, diabetes, smoking, and high cholesterol.

**Figure 2:**
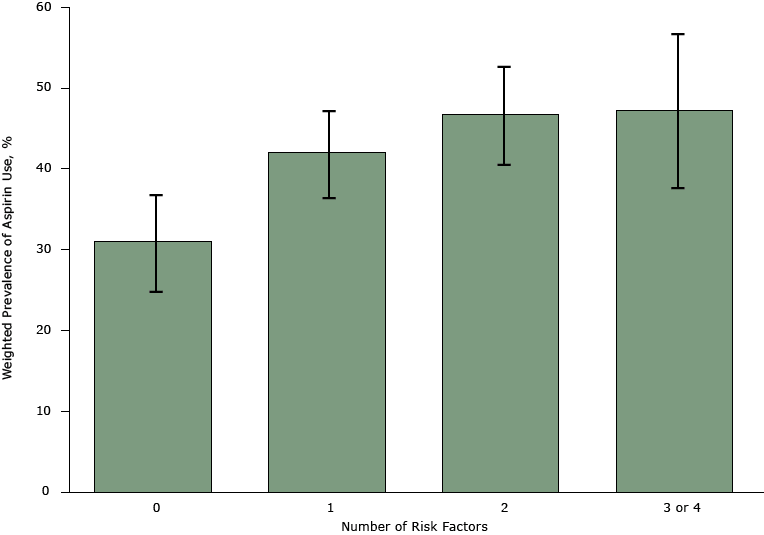
Weighted prevalence of aspirin use by number of myocardial infarction risk factors among men aged 45 to 79 years in North Carolina, Behavioral Risk Factor Surveillance System, 2013. Risk factors were hypertension, diabetes, smoking, and high cholesterol. Error bars represent 95% confidence intervals. **Table:** 

The prevalence of aspirin use among men with one, 2, or 3 or 4 risk factors was significantly greater than among those with no risk factors; however, we found no significant linear dose–response relationship between number of risk factors and aspirin use (*P* for trend = .25) (Figure 2). When risk of MI was dichotomized, the prevalence of aspirin use among respondents with one or more risk factors was 44.8% (95% CI, 41.0%–48.5%) and was significantly higher than the prevalence among respondents with no risk factors (prevalence ratio = 1.44; 95% CI, 1.17–1.78) (Table 2). Age was a positive predictor of aspirin use among respondents with one or more risk factors (Table 3).

No. of Risk Factors	Weighted Prevalence, % (95% Confidence Interval)
0	31.0 (25.0–37.0)
1	42.1 (36.7–47.6)
2	46.8 (40.8–52.8)
3 or 4	47.4 (37.8–56.9)

**Table 3:** Bivariate Analysis to Determine Predictors of Aspirin Use Among Men Aged 45 to 79 Years With at Least One Risk Factor for Myocardial Infarction in North Carolina (n = 1,182), BRFSS, 2013

Predictor	n^a^	Weighted^b^ Prevalence (%) of Aspirin Use (95% CI)	Prevalence Ratio (95% CI)	*P* Value^c^
**Age, y**				
45–54	334	38.8 (32.1–45.5)	1.0 [Reference]	.03
55–64	433	47.3 (41.5–53.2)	1.22 (0.98–1.50)	
65–74	324	52.2 (45.0–59.3)	1.34 (1.08–1.67)	
75–79	91	43.6 (30.7–56.6)	1.12 (0.80–1.58)	
**Race/ethnicity**				
White non-Hispanic	830	53.1 (46.7–59.5)	1.0 [Reference]	.22
Black non-Hispanic	205	47.8 (34.3–61.2)	0.90 (0.66–1.22)	
Hispanic	39	30.6 (9.0–52.1)	0.57 (0.28–1.17)	
Other non-Hispanic	96	58.5 (35.8–81.2)	1.10 (0.73–1.65)	
**Education**				
Less than high school	153	42.9 (29.0–56.7)	1.0 [Reference]	.81
High school	353	56.4 (46.8–66.0)	1.32 (0.91–1.89)	
More than high school	674	51.4 (44.0–58.8)	1.20 (0.84–1.71)	
**General health status**				
Fair or poor	272	47.2 (39.8–54.7)	1.0 [Reference]	.47
Good, very good, or excellent	905	44.1 (39.8–48.4)	0.93 (0.77–1.12)	
**Health insurance coverage**				
No coverage at all	141	36.2 (26.6–45.7)	1.0 [Reference]	.06
Some kind of coverage	1,036	46.2 (42.2–50.2)	1.28 (0.97–1.68)	
**Limited healthcare access due to cost**				
No	1,039	44.5 (40.5–48.5)	1.0 [Reference]	.71
Yes	139	46.5 (36.3–56.8)	1.05 (0.82–1.33)	
**Last routine medical checkup**				
A year or more ago	208	40.4 (32.1–48.7)	1.0 [Reference]	.23
Within the last 12 months	961	46.2 (42.0–50.4)	1.14 (0.91–1.43)	
**Body mass index, kg/m^2^**				
Normal weight (18.0–24.9)	215	43.2 (30.5–55.9)	1.0 [Reference]	.33
Overweight (25.0–29.9)	539	50.3 (42.3–58.2)	1.16 (0.83–1.62)	
Obese (≥30.0)	400	56.5 (47.3–65.8)	1.31 (0.93–1.83)	

Abbreviations: BRFSS, Behavioral Risk Factor Surveillance System; CI, confidence interval.

a Totals for some subcategories do not add to overall sample size (1,564) because of missing data

b Prevalence estimates are weighted to the overall population of men aged 45 to 79 in North Carolina using precalculated complex weight variables that are included in the BRFSS dataset.

c *P* value for Pearson design–based *F* statistic obtained from survey-weighted cross tabulation of aspirin use and potential predictors.

## Discussion

The USPSTF recommended aspirin use for the primary prevention of MI for men aged 45 to 79 years without contraindications to aspirin, when the benefits outweigh the risks in a context of patient–provider shared decision making. In our study sample, despite three-quarters of respondents having at least one risk factor for MI, the prevalence of aspirin use was below 50% both in the overall sample and among respondents with at least one risk factor. The prevalence rates of aspirin use in our study are slightly lower than those of another study, which found a 50% to 60% prevalence of aspirin use among men aged 45 to 79 in Oklahoma with risk factors for CVD (17). However, when compared with other studies that did not restrict their study populations to the sex and age groups for which aspirin may have benefits, our prevalence estimates are higher. For example, one study using BRFSS data from 24 states reported an adjusted prevalence of 32.7% for aspirin use among men and women aged 35 years or older (18). This finding suggests that reporting on aspirin use among the general adult population or among sex and age groups for which there is no clear evidence of the benefits, as is true for most studies using BRFSS data, understates the prevalence of aspirin use among populations that may benefit most from it. Even though the BRFSS cardiovascular health module may be administered to a broad age range of respondents, results should be reported specifically for groups for which there is evidence of the benefits of aspirin, such as men aged 45 to 79 years at risk for MI.

Our finding of low aspirin use among high-risk men also mirrors the findings of Mainous and colleagues (19). They analyzed National Health and Nutrition Examination Survey data to examine aspirin use for both primary and secondary prevention of CVD and found underutilization. This study and ours could benefit from having clinical data to determine the degree to which health care providers are recommending aspirin and to identify clinical decision-support tools that might increase aspirin use for appropriate high-risk patients (19).

Most respondents (87.8%) in our study sample said they were taking aspirin to prevent heart attacks, but 80% also said they were taking aspirin to prevent stroke. This finding suggests limited knowledge among study participants of the benefits of aspirin for various risk groups, such as those mentioned in the 2009 USPSTF recommendation. Health education interventions for both health care providers and patients may prove useful in increasing knowledge of the benefits and risks of aspirin. Improved knowledge may facilitate patient–provider shared decision making on aspirin. For our study, the MI risk profile of participants, in conjunction with the assertion of aspirin use, regardless of the intended use, is sufficient in determining the potential benefit of taking aspirin for MI prevention. Therefore, we used a yes response to the question “Do you take aspirin daily or every other day?” to define our primary outcome rather than a yes response to the question “Do you take aspirin to reduce the chance of a heart attack?”

Because most participants reported having health insurance and having a routine medical checkup in the previous year, health systems can play a key role in improving aspirin use in this population. High-risk respondents (those with at least one risk factor for MI) who had health insurance were significantly more likely to be taking aspirin than high-risk respondents who did not have insurance. This difference indicates that access to health care has a positive effect on population aspirin use. Consequently, more needs to be done to ensure that high-risk men see their health care providers for routine checkups, engage in patient–provider shared decision making, and use aspirin and other applicable components of the ABCS of heart disease and stroke prevention that are appropriate for their risk. A 2005–2008 study of the National Ambulatory Medical Care Survey and National Hospital Ambulatory Medical Care Survey found low prescribing rates for aspirin among physicians (46.9% of physicians prescribed aspirin for patients with ischemic vascular disease) and even lower prescribing rates in the South (37.1%) (20). With aspirin now included in the list of medications covered at no cost under the Affordable Care Act, shared patient–provider decision making may be facilitated, more providers may prescribe aspirin, and more high-risk patients may use it (21).

Although several strategies have been used successfully to increase aspirin use, community-based interventions have improved primary prevention efforts by bringing together primary care providers, public health professionals, and community resources (22,23). In a recent 16-month intervention in Hibbing, Minnesota, researchers used messaging on the use of aspirin for primary prevention to increase aspirin use (24). Working with primary care physicians in 3 health systems, they trained primary care health professionals and identified aspirin-eligible candidates to receive the messaging. Regular aspirin use increased from 36% at baseline to 54% at 4 months and 62% at 16 months. This combined public health–primary care approach may have the potential to improve aspirin use for primary prevention.

Our study has several limitations. The results are limited by the inherent bias associated with self-reported survey data. Also, as a result of methods changes in BRFSS in 2011 (25), we cannot compare aspirin use before and after the USPSTF recommendation. Furthermore, we could not more precisely quantify and stratify risk of MI among participants because the BRFSS lacks clinical and laboratory data on blood pressure and cholesterol levels that would be needed for such calculations. Instead, we defined high risk of MI simply by a self-reported history of diabetes, high cholesterol, high blood pressure, or current smoking. Finally, the strongly skewed distribution of respondents across categories of potential predictors limited our ability to detect statistically significant effects during bivariate and multivariate analyses.

The administration of the cardiovascular health module to a split sample (about 50%) of the eligible BRFSS respondent population had the potential to limit our study sample size. However, we determined that our outcome of interest was missing at random among nonrespondents of the cardiovascular health module. This finding allowed us to impute missing responses and recover data on respondents that would have otherwise been lost to analysis (14). Notwithstanding our success in overcoming this potential limitation, split-sample questionnaire administration is a limiting factor to the sample size of all potential studies that rely on noncore BRFSS modules. In addition, strict statistical conditions (missing at random and missing completely at random) must be met before imputation can be performed with minimal bias, and imputation could be a statistically complex process (14). Therefore, to eliminate this potential limitation, it may be worth considering administering the cardiovascular health module (and potentially all other noncore modules) to the entire BRFSS sample rather than to a split sample in future years.

Bertoni and colleagues estimated that North Carolina must have 30,000 fewer cases to meet the national Million Hearts objective of preventing one million heart attacks and strokes by 2017 (26,27). Given the low aspirin use in our study sample and the effectiveness of aspirin in preventing MI among some men, increasing aspirin use among high-risk men may be a useful component toward achieving the Million Hearts goal for North Carolina.

## Appendix A Sample Size Scenarios With and Without Imputation of Missing Outcomes, North Carolina Behavioral Risk Factor Surveillance System, 2013

**Table:**

Characteristic	No Imputation	Imputation
Eligible study population (all male respondents aged 45–79 y)	2,094	2,094
Nonrespondents to cardiovascular health module (no data on outcome, ie, aspirin use)	−1,163	0
Initial study population (respondents with data on aspirin use)	931	2,094
Exclusion criteria no. 1: contraindication to aspirin	−74	−163
Exclusion criteria no. 2: history of cardiovascular disease	−166	−367
Final study sample	691	1,564
At risk for myocardial infarction^a^	518	1,182

^a ^The primary focus of the study was aspirin use among men at risk of myocardial infarction; therefore, most of the analyses were done on this subset of the study sample.

## Appendix B Frequency Distribution of Key Variables Among Respondents and Nonrespondents of the Cardiovascular Health Module, North Carolina Behavioral Risk Factor Surveillance System, 2013

**Table:**

Covariates	Cardiovascular Health Module	
	Respondents, %	Nonrespondents, %
**Hypertension**		
Yes	45.6	44.2
No	54.4	55.8
**Diabetes**		
Yes	15.1	14.9
No	84.9	85.1
**High cholesterol**		
Yes	46.5	45.9
No	53.5	54.1
**Current smoking**		
Yes	17.3	18.1
No	82.7	81.9
**Sex **		
Female	62.2	60.0
Male	37.8	40.0
**Age, y**		
<45	26.9	30.7
45–64	38.3	37.0
>64	34.8	32.3
**Race/ethnicity**		
White	68.1	68.2
Black	19.2	18.9
Hispanic	5.2	5.2
Other	7.5	7.7
**Education**		
<High school	13.5	16.5
High school	28.7	27.7
>High school	57.8	58.8
**Health status**		
Fair or poor	23.1	22.1
Good, very good or excellent	76.9	77.9
**Health insurance coverage**		
No coverage at all	14.7	14.9
Some kind of coverage	85.3	85.1
**Limited health care access due to cost**		
No	84.2	83.5
Yes	15.8	16.5
**Last routine medical checkup**		
A year or more ago	19.8	21.9
Within the last 12 months	80.2	78.1
**Body mass index, kg/m^2^**		
Normal weight (18.0 to 24.9)	30.9	33.3
Overweight (25.0 to 29.9)	37.5	35.6
Obese (≥30.0)	31.6	31.1
